# Knowledge of Cervical Cancer, Human Papilloma Virus (HPV) and HPV Vaccination Among Women in Northeast China

**DOI:** 10.1007/s13187-019-01582-7

**Published:** 2019-07-24

**Authors:** Yu-e Ning, Yao Liu, Xiao-yu Xu, Xin-yu Zhang, Ning Wang, Li-qiang Zheng

**Affiliations:** Department of Obstetrics and Gynecology, Shengjing Hospital of China Medical University, Shenyang, 110020 China

**Keywords:** Cervical cancer, Screening, HPV, Vaccinations, Knowledge and acceptability

## Abstract

This study aimed to research the understanding and knowledge of cervical cancer, human papilloma virus (HPV), and HPV vaccination, and the acceptance of HPV vaccination, among a population of women in northeastern China. A cross-sectional survey was carried out by questionnaire to investigate knowledge of cervical cancer, HPV, and HPV vaccination. The 230 female participants were native residents of northeastern China, and their ages ranged between 18 and 65 years. Questionnaires were randomly acquired by the respondents from online and paper questionnaire distribution. The questionnaire included questions on three major aspects to record people’s perceptions of cervical cancer, HPV, and vaccines. Of the sample of 230 women surveyed, 80.9% had heard of cervical cancer, but understanding was only 15.7%; 38.3% knew about HPV; 20% knew about HPV vaccine; 39.6% agreed to receive HPV vaccination, and the remainder were mainly concerned about its safety and effectiveness. Data analysis showed that age, family income, and whether there was experience of screening all influenced knowledge of cervical cancer, but this was not statistically significant. The level of education had no obvious effect on the degree of knowledge about cervical cancer; however, with an improvement in education, women’s awareness of HPV vaccine improved significantly (*p* < 0.05). Women who have received cervical cancer screening had significantly greater knowledge about cervical cancer and HPV than those with no screening (*p* < 0.05). Women in northeastern China have little knowledge of cervical cancer, HPV, and HPV vaccine, lack disease knowledge, and hold a skeptical attitude about HPV vaccination. Medical institutions are the main channel providing information to these women.

Cervical cancer (CC) is the third highest cause of cancer-related death for females in low- and middle-income countries [1, 2]. Over the past 10 years, the incidence of CC in developed countries has decreased, while the incidence in developing countries and in young women is on the rise, which is a serious threat to women’s health [3, 4]. Sustained infection with high-risk HPV is a prerequisite for CC and precancerous lesions, causing more than 90% of such lesions. Research on vaccination has increased in recent years. Population surveys regarding CC, human papilloma virus (HPV), and associated issues have been carried out in many countries [2, 5, 6]. Though in China there is a clear policy about cervical cancer screening, we still do not have an explicit policy on HPV vaccine and vaccination. Data and evidence of the knowledge and awareness of HPV infection, cervical cancer, and HPV vaccine remain unclear. Investigations involving the knowledge about CC and HPV vaccine in different regions and populations have been carried out recently in China, but the majority of the studies have been conducted in the southern provinces. There have been few studies on knowledge of CC and HPV in northeastern China, and large-sample studies to identify factors affecting the knowledge of CC and HPV in the population, or to provide potential solutions, are lacking. A variety of factors including region, ethnic group, culture, custom, and economic development level can affect the knowledge of CC and HPV in a population. At the same time, China’s HPV vaccination policy and vaccination costs will also affect the promotion of vaccines. In this study, we conducted a questionnaire survey regarding CC, HPV infection, and HPV vaccination among women in northeastern China in order to understand the differences in the knowledge of these issues among the women, their concerns about accepting HPV vaccination, as well as the barriers impeding the promotion of the HPV vaccine in this region.

## Materials and Methods

### Survey Participants

A total of 400 persons, including hospital visitors and outpatients without cervical disease or relevant history, were treated in the Nanhu Branch and Huaxiang Branch of the Shengjing Hospital between October 2015 and April 2016. In addition, on-line participants were included in the study (300 completed paper questionnaires and 100 online questionnaires were received). Inclusion criteria are as follows: women aged 18–65 years; native to the three northeastern provinces (Liaoning, Jilin and Heilongjiang); questionnaire completion rate > 95%. Exclusion criteria are as follows: age < 18 years old or > 65 years old; not native to the three northeastern provinces; questionnaire completion rate < 95%; cannot complete the questionnaire independently due to low educational level or other conditions.

### Survey Methods

(1) Some participants completed the hard-copy of the questionnaire on-site; (2) online questionnaires were distributed through the mobile app called WeChat, and the participants completed the questionnaire using their smartphone and then submitted it; (3) each questionnaire was coded and the date were recorded to ensure authenticity, validity, and completeness.

### Questionnaire Design

The study used a questionnaire survey. The questionnaire was designed on the basis of the cultural background, customs, cognitive channels, and other factors in northeastern China. A large number of domestic and international survey studies were also referenced during the design of the questionnaire. The instrument consists of three major parts. The first part of the survey collected demographic information. The first part enquires about the basic situation of the women and obtains basic information about the participants, such as their native place, age, fertility history, and gynecological examination history.

The second part investigates participants’ awareness and sources of information of CC. This part includes, have you heard of cervical cancer? Have you heard of precancerous lesions of the cervix? The high age of cervical cancer? What is the frequency of screening for cervical cancer? Etc (Table 2). After descriptive analysis, those who answered “yes” to these questions were assigned 1 point, while those who answered “no” were assigned 0 points, in order to assess the relationship between women in NE China and CC knowledge. There is a total of 6 points: participants achieving 0–3 points were considered to have no knowledge of the disease and those with 4–6 points had certain knowledge of the disease.

The third part investigated knowledge of HPV and HPV vaccines. These items included whether the participant had heard of HPV, how they had heard about HPV, HPV virus transmission route, whether they had heard of HPV vaccine, how they had heard about HPV vaccine, and whether the HPV vaccine can prevent CC. The following questions were raised regarding the acceptance of HPV vaccines: acceptance of HPV vaccine, acceptable price, acceptable vaccination age, vaccination interval, etc. All items were scored quantitatively. After descriptive analysis, these responses were dichotomized as “correct” or “yes” (1 point) and “incorrect” or “do not know” or “no”(0 point). The total number of points available was 10. Achieving less than 1/3 of the total points (0–3) indicated that the participants had no knowledge of HPV; 1/3–2/3 of the total points (4–7 points) represented moderate knowledge and more than 2/3 (8–10 points) represented full knowledge.

### Statistical Methods

Epidata 3.1(http://www.epidata.dk/) was used to input the data. Dual data entry and consistency testing were applied to ensure the accuracy and reliability of the data. All data were analyzed using SPSS18.0 software (IBM, Armonk, NY, USA). Measurement data were expressed as mean ± SD, enumeration data were expressed as rates, and three groups of measurement data were analyzed by one-way analysis of variance (one-way ANOVA). The enumeration data were subjected to the *χ*^2^ test or Fisher’s exact test. *p* < 0.05 indicated a statistical significance.

## Results

### General Information

A total of 300 hard-copy questionnaires were handed out and 205 were returned, including 157 valid questionnaires (validity rate 76.6%); 100 on-line questionnaires were returned, including 73 valid questionnaires (validity rate 73%). There were a total of 230 valid questionnaires and the overall validity rate was 75.4%. The age range of the respondents was from 19 to 68 years old (39.8 ± 11.8 years old) (mean ± SD). Liaoning province accounted for 45% (*n* = 103, average age 39.8 ± 12.2 years of the respondents); Jilin province accounted for 29% (*n* = 67, average age 40.3 ± 12.1 years); and Heilongjiang province accounted for 26% (*n* = 60, average age 39.8 ± 11.1 years). There was no significant difference in the age of the respondents among the three provinces (Table 1).

**Table 1:** Age distribution of the participants in the three provinces

Province	*n* (230)	Age (years)*
Liaoning	103	39.8 ± 12.2
Jilin	67	40.3 ± 12.1
Heilongjiang	60	39.2 ± 11.1
P	–	0.885

*Mean ± SD

### Knowledge of CC and CC Screening

**Fig. 1:**
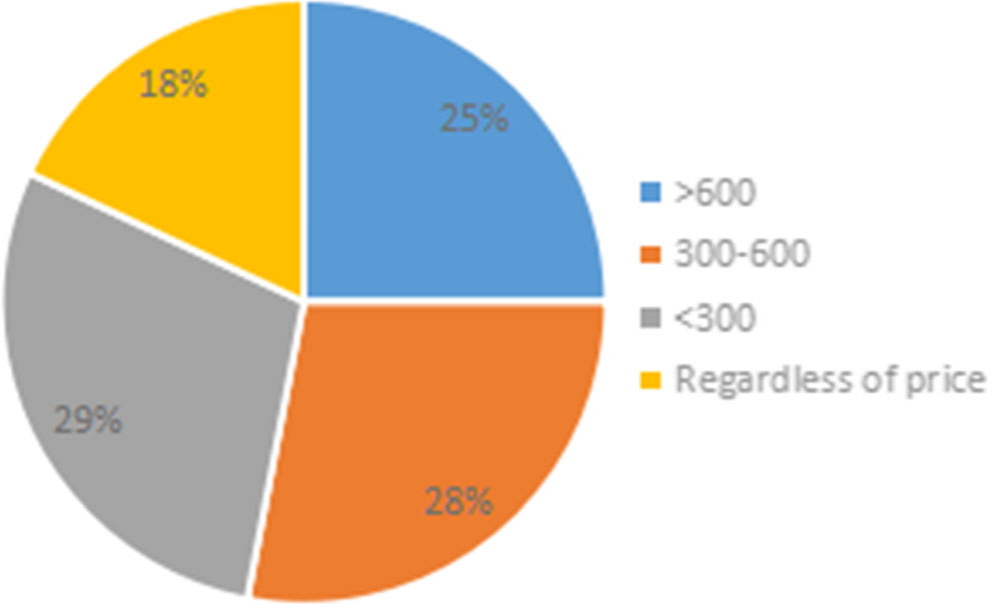
Price of CC screening

The overall awareness rate of CC among the women in the three northeastern provinces was 15.7%. Although it was highest in Heilongjiang province (18.3%) compared with Liaoning province (14.6%) and Jilin province (14.9%), the difference was not statistically significant (*p* = 0.8). Urban residents showed slightly greater knowledge of CC than rural residents but the difference was also not statistically significant (*p* = 0.48). Additionally, educational level did not significantly contribute to the differences in the degree of knowledge (*p* = 0.97). With an increase in family income, the knowledge of CC increased accordingly: it was highest in women with family annual income of 110–200 thousand yuan; however, the difference in the knowledge of CC among the women with different levels of family income did not achieve statistical significance (*p* = 0.06). The results showed that marital status, the number of sexual partners, and a history of gynecological examination did not significantly affect the knowledge of CC (*p* = 0.827/0.981/0.42). Only 30% of the participants had once received CC screening. The women who had received CC screening had significantly greater knowledge of the disease than those who had never undergone screening (*p* = 0.002) (Table 2). Regarding the cost of the screening test, which was acceptable to the women in the three provinces, 29% of the women investigated thought the price should be less than 300 RMB (about $44), 28% of them could accept a price of 300–600 RMB, and 25% could accept a price of more than 600 RMB. Another 18% of women would not take price into account (Fig. 1, Table 2).

**Table 2:** Knowledge of CC among women in the three northeastern provinces of China

	Knowledge (percentage)	No knowledge	*χ*^2^	*p*
Province				
Liaoning	15 (14.6%)	88	0.45	0.8
Jilin	10 (14.9%)	57		
Heilongjiang	11 (18.3%)	49		
District				
Urban	27 (16.8%)	134	0.51	0.48
Rural	9 (13.0%)	60		
Educational level				
Junior high school or below	9 (15.3%)	50	0.07	0.97
Senior high school or college	13 (16.9%)	64		
University or above	14 (16.5%)	71		
Unfilled	0 (0.0%)	9		
Age				
18–30	7 (11.1%)	56	3.86	0.28
31–40	6 (16.2%)	31		
41–50	2 (7.7%)	24		
> 50	21 (20.2%)	83		
Yearly family income (RMB (Renminbi))				
10,000–50,000	16 (13.0%)	107	7.4	0.06
60,000–10,0000	12 (15.2%)	67		
110,000–200,000	6 (40.0%)	9		
> 200,000	2 (15.4%)	11		
Marital status				
Unmarried	8 (17.0%)	39	1.022	0.827
Married	27 (15.9%)	143		
Divorced	0 (0.0%)	7		
Widowed	1 (16.7%)	5		
Number of sexual partners				
0	3 (13.6%)	19	0.392	0.981
1–2	28 (15.6%)	151		
≥ 3	2 (18.2%)	9		
Unfilled	3 (16.7%)	15		
Whether they had received gynecological examination				
Yes	30 (18.2%)	135	4.13	0.42
No	5 (8.6%)	53		
Unfilled	1 (14.3%)	6		
Expected vaccination age				
< 10	7 (11.1%)	56	3.86	0.28
10–15	6 (16.2%)	31		
16–18	2 (7.7%)	24		
> 18	21 (20.2%)	83		
Whether had received CC screening				
Yes	19 (27.5%)	50	9.58	0.002**
No	18 (11.2%)	143		
Whether it is recommended to vaccinate adolescents				
Yes	20 (16.0%)	105	0.03	0.87
No	16 (15.2%)	89		
Whether they hope HPV vaccine will be listed in national immunization plan				
Yes	34 (18.4%)	150	5.52	0.02**
No	2 (4.3%)	44		

**p < 0.05

### Knowledge of HPV Among the Women in the Three Provinces

**Fig. 2:**
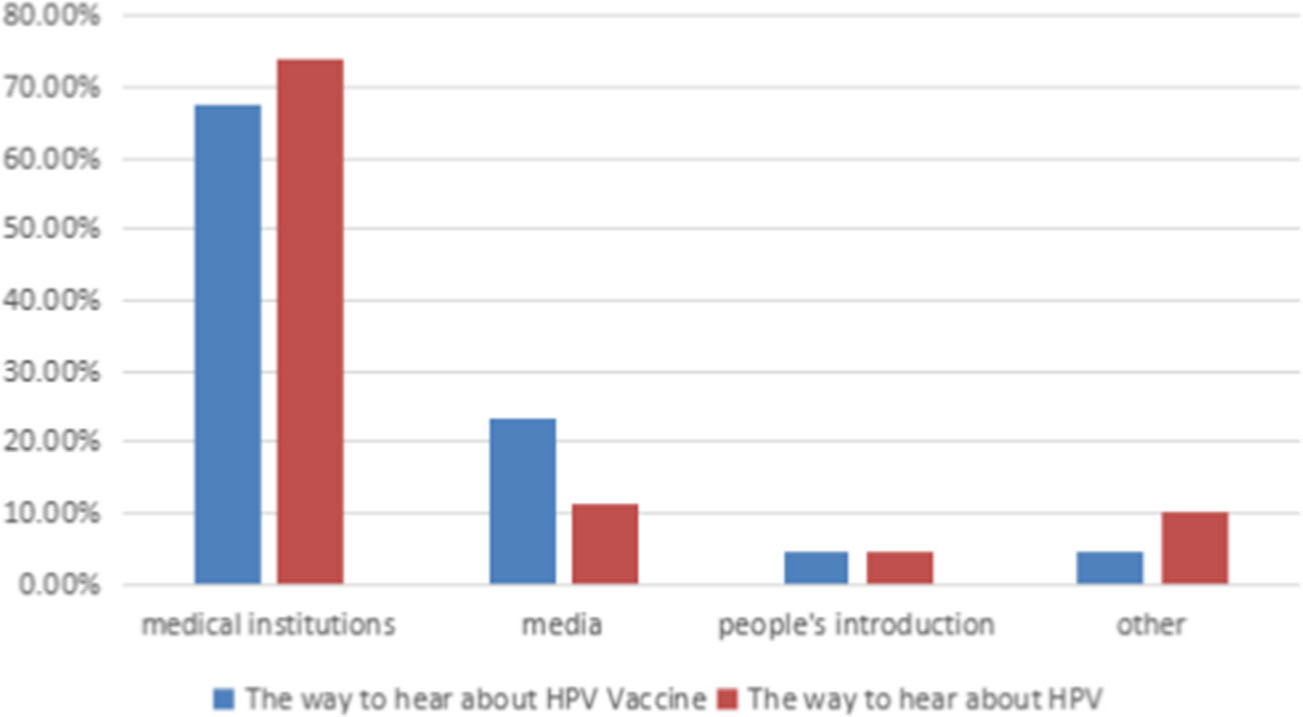
Sources of information about HPV and HPV vaccine

Our results showed that, among the women in the three northeastern provinces, the percentage of respondents who had full knowledge of HPV was 4.3%, moderate knowledge accounted for 18.6%, and no knowledge accounted for 77.0%. The women in Liaoning province had a slightly higher level of knowledge than those in Heilongjiang province, although the difference was not statistically significant (*p* = 0.77), suggesting the knowledge of HPV among the women in northeastern China is independent of region. Educational level was associated with knowledge of HPV. The respondents with a high level of education had greater knowledge of HPV and HPV vaccine (*p* < 0.05). Moreover, age was also associated with the knowledge of HPV. Women in the age range of 31–40 years had greater knowledge of HPV than women in other age ranges; women who were older than 50 years old had the lowest level of knowledge of HPV (*p* = 0.003). Other factors, including living environment (urban or rural), the number of sexual partners, marital status, and gynecological examination history, did not show a significant association with knowledge of HPV (*p* = 0.34/0.81/0.52/0.09). Among the women investigated, 38% (*n* = 88) had heard of HPV: 74% of these women had learned about it from medical institutions, and 11% and 5% had learned of it from the media or from friends or relatives, respectively (Fig. 2, Table 3).

**Table 3:** Knowledge of HPV among women in three northeastern provinces of China

	Full knowledge (percentage)	Moderate knowledge (percentage)	No knowledge	*χ*^2^	*p*
Province					
Liaoning	7 (6.8%)	20 (19.4%)	76		
Jilin	4 (6.0%)	10 (14.9%)	53	1.83	0.77
Heilongjiang	2 (3.3%)	9 (15.0%)	49		
District					
Urban	8 (5.0%)	34 (21.1%)	119	6.76	0.34
Rural	5 (7.2%)	5 (7.2%)	59		
Educational Level					
Junior high school or below	6 (10.2%)	3 (5.1%)	50	40.72	< 0.001**
Senior high school or college	1 (1.3%)	4 (5.2%)	72		
University or above	6 (7.1%)	27 (31.8%)	52		
Unfilled	0 (0.0%)	5 (55.6%)	4		
Age					
18–30	4 (5.8%)	13 (18.8%)	52	18.34	0.003**
31–40	6 (12.2%)	12 (24.5%)	31		
41–50	2(3.1%)	13(20.0%)	50		
> 50	1 (2.1%)	1 (2.1%)	45		
Yearly family income (RMB)					
10,000–50,000	4 (3.3%)	18 (14.6%)	101	7.792	0.201
60,000–100,000	6 (7.8%)	17 (22.1%)	54		
110,000–200,000	2 (11.8%)	4 (23.5%)	11		
> 200,000	1 (7.7%)	1 (7.7%)	11		
Marital status					
Unmarried	2 (4.3%)	11 (23.4%)	34	5.16	0.52
Married	11 (6.5%)	27 (15.9%)	132		
Divorced	0 (0.0%)	0 (0.0%)	7		
Widowed	0 (0.0%)	1 (16.7%)	5		
Number of sexual partners					
0	1 (4.5%)	4 (18.2%)	17	3.03	0.81
1–2	11 (6.1%)	30 (16.8%)	138		
≥ 3	1 (9.1%)	3 (27.3%)	7		
Unfilled	0 (0.0%)	2 (11.1%)	16		
Whether they had received gynecological examination					
Yes	12 (7.3%)	31 (18.9%)	122	4.77	0.09
No	1 (1.5%)	8 (12.3%)	56		
Expected vaccination age					
< 10	3 (4.8%)	6 (9.7%)	53	9.938	0.103
10–15	2 (5.3%)	7 (18.4%)	29		
16–18	4 (15.4%)	7 (26.9%)	15		
> 18	4 (3.8%)	19 (18.3%)	81		
Whether they had received CC screening					
Yes	4 (5.8%)	22 (31.9%)	43	17.51	< 0.001**
No	9 (5.3%)	17 (10.0%)	144		
Whether they accept HPV vaccination					
Acceptable	6 (6.6%)	21 (23.1%)	64	5.81	0.21
Unacceptable	2 (3.1%)	10 (15.6%)	52		
No choice	5 (6.7%)	8 (10.7%)	62		
Whether it is recommended to vaccinate adolescents					
Yes	8 (6.4%)	24 (19.2%)	93	1.4	0.497
No	5 (4.8%)	15 (14.3%)	85		
Whether they hope HPV vaccine will be listed in national immunization plan					
Yes	12 (6.2%)	37 (19.2%)	144	5.185	0.054
No	1 (2.7%)	2 (5.4%)	34		

**p < 0.05

### Knowledge About and Attitudes to HPV Vaccine

**Fig. 3:**
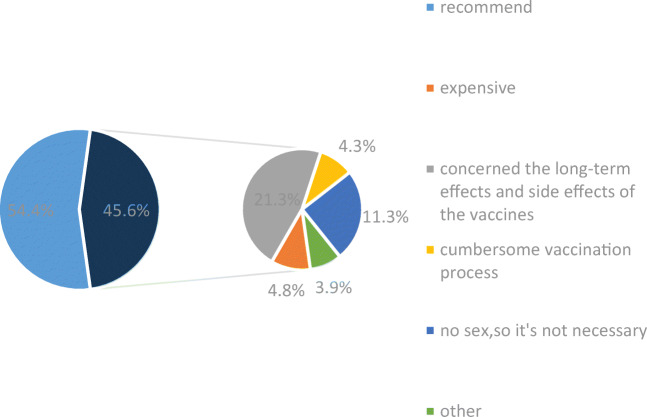
Whether adolescents should receive the HPV vaccine and reasons for not recommending

In the population surveyed, 20% (*n* = 46) of women had heard of HPV vaccine; 43 respondents provided information on the channels through which they knew about HPV vaccine: 67% (*n* = 29) learned it from medical institutions, 23% (*n* = 10) from mass media, 5% (*n* = 2) from friends, and 5% (*n* = 2) from other channels (Fig. 2). Regarding whether the vaccine was acceptable, 39.6% (*n* = 91) of women chose “acceptable”, 27.8% (*n* = 64) held a negative attitude, and 32.6% (*n* = 75) held a “wait-and-see” attitude and did not make a choice. Notably, among the women surveyed, none had received HPV vaccination. Although the HPV vaccine had been introduced 10 years previously, the vaccination rate in this region was extremely low. According to our survey, 54.4% respondents agreed with the recommendation of vaccination in adolescents, 45.6% respondents disagreed with the recommendation of vaccination in adolescents, and 21.3% of the respondents were concerned about the long-term effects and side effects of the vaccine (Fig. 3). In total, 84% of respondents (*n* = 193) hoped that HPV vaccine would be listed in the national immunization plan. The women who had a high knowledge of CC were more likely to hope that HPV vaccine would be included in the national immunization plan, although the association was not statistically significant.

## Discussion

In 2013, the American Cancer Society (ACS) reported that 4030 patients died of CC among the 12,340 with newly diagnosed CC. The incidence of squamous cell carcinoma, which is sensitive to radiotherapy and has a good prognosis, is gradually decreasing, but the incidence of adenocarcinoma is increasing. In particular, the incidence of CC in younger women shows an increasing trend which is more obvious in developing countries. In China, the incidence of CC is second only to that of breast cancer as the disease which most seriously threatens women’s health. Since 2004, the Chinese government has been actively promoting and carrying out a CC screening program, including establishing pilot and demonstration sites for early intervention in patients with CC. Up to 2009, CC pilot sites had been established in 221 counties in 31 provinces or autonomous regions. Large-scale education and screening have significantly decreased the incidence and mortality of CC in China [7]. However, owing to a huge population base, the limited economic developmental level, unbalanced allocation of healthcare resources, and the low knowledge of the disease among Chinese women, China still lags behind developed countries in the prevention of CC. Notably, the incidence of CC in China is six times than that in developed countries and Chinese CC cases account for one-third of the cases in the world. Meanwhile, the incidence of the disease increases by 2–3% annually in China and every day about 100 Chinese women die of CC [4].

Fortunately, CC has a good prognosis if it can be screened, detected, and treated early. In this study, 43% of women said they could accept the current price of CC screening, and those who had undergone CC screening had higher recognition of CC and HPV than women who had not had CC screening. Therefore, the price of CC screening plays an important role in women’s awareness of CC and HPV. The price of CC screening varies among hospitals. This study hopes to provide some data to guide the pricing policy for CC screening. Appropriately reducing the price of CC screening or including it in outpatient health insurance coverage would allow more people to have the opportunity to undergo CC screening. At the same time, promoting relevant knowledge from all aspects will ensure that most people understand the screening and regularly attend a hospital for examination. There is a long way to go to achieve universal coverage of CC screening in China.

In 1976, Harald Zur Hausen put forward the association between HPV and CC for the first time [8]; this encouraged the study of the pathogenesis of CC. There are over 100 genotypes of HPV which are classified into two groups according to their ability to transform epithelial cells: high risk and low risk. In the current survey, we found that 77% of women in the three northeastern provinces did not understand the link between HPV infection being a precursor for developing cervical cancer. This percentage is slightly higher than that reported by Shenzhen and Macau [9, 10], which may be related to local economic level, education base, geographic location, health awareness, and culture. Shenzhen, Macao, and other regions have a fast pace of life, and people pay more attention to their health under the prevailing conditions of high intensity and high pressure. At the same time, the understanding of HPV vaccine was similar to that of CC. Two-thirds of women surveyed obtained their information from medical institutions, which re-emphasizes the importance of medical institutions in conveying knowledge. The findings of this study are similar to those of a recent study. More people choose to believe in doctors, and the online platform also shows advantages. Therefore, a variety of publicity strategies can be combined to promote the HPV vaccination rate and promote HPV prevention and control of related diseases [11–14]. At the time of treatment, doctors should strengthen the promotion of CC and related knowledge. Medical colleges should provide teachers and students with relevant knowledge to inform the public, and also promote mass communication through the power of the media. Schools should also introduce relevant knowledge to improve people’s understanding of CC and HPV.

Women are not only susceptible to HPV infection, the cause of CC, but are also the guardians of children. They play a decisive role in whether or not to vaccinate. Parents’ knowledge of HPV and health awareness directly affects their willingness to vaccinate their children. Therefore, it is of great significance to investigate women’s awareness of CC and HPV, and of the corresponding vaccine [5, 10]. In the population surveyed, 20% (*n* = 46) of women had heard of HPV vaccine. This finding is in line with a study reported in 2017 in which the rate of awareness of HPV vaccine was 17.13% [15]. The survey found that 45.6% (*n* = 105) of women disagreed to recommend HPV vaccine to adolescents. The reason why vaccination is not recommended for adolescents is mainly concerned with the long-term effects and side-effects of the vaccine. It has been reported in the literature that about 6–12% of parents in Europe and the USA worry that vaccination will promote premature sexual behavior among adolescents [16, 17]. In this study, 54.4% (*n* = 125) of women agreed to recommend HPV vaccine to adolescents, but nearly half of the women considered that the most suitable age for vaccination was over 18 years old. The American Academy of Pediatrics (AAP) recommends that HPV vaccination should be performed at 11–12 years old for both boys and girls since HPV vaccines are most effective before adolescents begin sexual activity [12]. HPV vaccines have been launched in most countries and regions of the world; more than 80 countries have listed HPV vaccines in their national vaccination program, and 30 countries or regions have listed them in their pilot vaccination program. However, China is still in a state of voluntary vaccination. In our survey, 83.9% of women hoped that HPV vaccination would be incorporated into the national immunization plan, stating that if HPV vaccination becomes routine, they would agree to their daughters receiving HPV vaccine. Therefore, China should also include HPV vaccines in national vaccination programs as soon as possible to increase the vaccination rate among adults and appropriate children, in order to reduce the trend toward a higher incidence of CC in the young.

The price of the HPV vaccines launched is about RMB1000/dose and it is recommended to use three doses. In our survey, few of the women investigated could accept such a price. The price which could be accepted by most respondents was below RMB500. Manufacturers should consider speeding up vaccine development, reducing vaccine prices to make them affordable to more people, increasing the number of vaccines, and enabling people to be vaccinated in order to greatly reduce CC incidence. In addition, the HPV vaccine should be included in the scope of medical insurance reimbursement, and the reimbursement rate should be increased as much as possible. In recent years, some provinces and cities in southern China have included it in the scope of medical insurance reimbursement, but, owing to the limited number of vaccines and large population, it is difficult to popularize each one that is suitable for women. In the procurement of vaccines, the health department has signed a procurement agreement to ensure the supply of future products and to reduce the sales price as much as possible [17]. Given the short-term listing of vaccines in mainland China, it is difficult to make an appointment. Instead, one may need to go to Hong Kong or abroad to be vaccinated, incur high costs, and may face the risk of vaccines becoming out of stock, which are reasons behind the low vaccination uptake by women in northeastern China [18].

Current HPV vaccines include prophylactic and therapeutic vaccines. Therapeutic HPV vaccines are still being investigated. Most HPV vaccines, including those launched in China, are prophylactic vaccines. There are no reports of serious adverse reactions so far [13]. However, HPV vaccines cannot prevent infections by all types of HPV. Although prophylactic HPV vaccines are essential for the primary prevention of CC, for those who have been vaccinated with HPV vaccines, CC screening is still required [2]. With the increase in the incidence of CC, it is important to improve the knowledge of CC and HPV among women [5, 19], to minimize the price of CC screening and HPV vaccines, and to include them in the scope of health insurance, so that most people have the ability to receive CC screening and to afford HPV vaccine. This would eliminate CC.

Many studies in China and abroad have shown that people’s awareness of CC, HPV, and its vaccine can effectively improve the vaccination rate of HPV [5, 6, 9], reduce the incidence of CC, and the mechanism of action, effectiveness, and safety of vaccines. Where the HPV vaccination rate is higher, the mortality rate of CC is lower. This study found that two-thirds of women’s understanding of CC in northeastern China comes from medical institutions. Therefore, effective measures to improve the level of knowledge of HPV include strengthening the publicity and education provided by medical institutions, especially in the era of network information, news websites, and social media platforms. The online medical service platform and knowledge base are developing rapidly. A professional platform should be added to disseminate relevant knowledge on a regular basis, so that people can quickly and comprehensively increase their knowledge of HPV and HPV vaccines [5, 20, 21].

At present, studies involved in disseminating knowledge of CC and HPV in northeastern China, particularly large-sample studies to identify the reasons for the low knowledge of the disease in this region, as well as providing potential solutions, are still lacking. To improve knowledge of the disease among the women of this region and increase the HPV vaccination rate, on the one hand, requires appropriate education and follow-up from medical staff, and publicity via the media or internet. On the other hand, the support of vaccine suppliers is important, for example, in developing low-price vaccines and launching corresponding policy incentives [2, 21]. In addition, factors such as family income, education level, culture, and conservative attitudes influence women’s awareness of CC in northeastern China. Finally, the strong support of the government is particularly important; measures such as increasing the number of vaccination clinics, reducing the vaccine cost, and increasing the coverage of healthcare insurance can effectively improve the popularization and application of HPV vaccine.

## Conclusion

The survey showed that women in northeastern China had a low level of knowledge of CC, HPV infection, and HPV vaccine; 83.4% of the women lacked knowledge of the disease and had a skeptical attitude toward HPV vaccine. It is important to strengthen the knowledge of the disease and HPV vaccine, and to increase the HPV vaccination rate among the women in this region. Medical institutions are the primary channel used by the women in this region to acquire relevant information; therefore, enhancing the education and propaganda offered by medical institutions is an important avenue by which to prevent CC. Meanwhile, reducing the HPV vaccine price and increasing its coverage are crucial for improving HPV vaccination rates.
